# Conundrum between internationalisation and interdisciplinarity: reflection on the development of medical humanities in Hong Kong, Taiwan and China

**DOI:** 10.15694/mep.2018.0000184.1

**Published:** 2018-09-03

**Authors:** Harry Yi-Jui Wu, Julie Y. Chen

**Affiliations:** 1 The University of Hong Kong

**Keywords:** medical humanities, interdisciplinarity, internationalisation

## Abstract

This article was migrated. The article was marked as recommended.

This article reviews the development of medical humanities pedagogies in Taiwan, China and Hong Kong. We reflect on the curricula formation and implementation regarding their interdisciplinary nature and point out the challenges educators face under the climate of current university practices. We first indicate that the emergence of medical humanities in the three societies was enabled by various social forces across the Strait. It also depended on opportunities offered by the higher education reform. We then provide a detailed experience of interdisciplinary team building at The University of Hong Kong, followed by a critical reflection on the challenges of medical humanities along the pursuit of internationalisation among universities in three Chinese societies. We find that the clashing objectives under universities’ strategic planning framework could lead to changes in work environments and research practices, hampering the design and the delivery of the curricula. In the end, the idealised promise of the interdisciplinarity of such curricula could become fugacious.

## Interdisciplinarity of medical humanities

Cited by educators in various higher education institutions in Hong Kong, China and Taiwan, medical humanities is characterised by an interdisciplinary pedagogy by nature ( Tsui et al, 2008; Chen et al, 2017; Yun et al, 2017). Whilst medical humanities as a pedagogical initiative was shaped roughly in the 1960s ( Fox, 1985), most of the medical humanities programmes in the universities using Chinese as the medium of instruction were developed in the early twenty-first century. They define medical humanities in various ways. Whilst no clear definition of the scholarship is available, most of them refer to the definition of medical humanities developed in the Anglo-American context. For example, in the higher education institutions in Taiwan and Hong Kong and some in China, the most commonly cited definition of medical humanities is the mission statement propagated by New York University. The statement defines medical humanities as a concept that aims ‘to include an interdisciplinary field of humanities (literature, philosophy, ethics, history and religion), social science (anthropology, cultural studies, psychology, sociology), and the arts (literature, theater, film, multimedia and visual arts) and their application to healthcare education and practice’ ( LitMed NYU, 1993).

Over the past decade, it has been proven that teaching and learning activities in medical humanities are useful in cultivating students’ reflective thinking, perspective taking and observational skills. However, challenges remain immense, given the evolving nature of medical education and practice. One of the most salient concerns dwells on the interdisciplinarity of the initiative. Due to the changing nature of care in response to chronic diseases, as well as the evolving sites where care is delivered, scholars have called for a higher level of interdisciplinarity and more inclusive and wider applications of the initiative because the key sites for care provision and idea generation are now outside medicine. The term ‘health humanities’ can better capture the landscapes of care that are concerned about not only diseases but also actual lived experiences and patients’ overall health condition and well-being ( Jones et al, 2017).

In medical education, however, it is still debatable whether disciplines should work independently or in concert with others to achieve a synthesised goal ( Wear, 2009). Whilst the types and the levels of interdisciplinarity in the work of medical humanities remain unassessed, over the last decade, more humanities scholars and social scientists have joined the faculties of medical schools. Despite the advantage of enriching methods and styles of research and teaching, the vision of interdisciplinarity has not always been promising. It has been reported that scholars have to alter their research practices to fit in their newly acquired identities. They no longer conduct independent slow and painstaking research. Instead, they have to join the scientists or clinicians to publish shorter and higher-impact articles. This is because the work environment in most medical schools largely does not deliver on the promise of inclusiveness ( Albert et al, 2015). Another noteworthy point is that whilst interdisciplinary research creates innovation, it requires team members of different disciplines to learn the language they are originally unfamiliar with in order to talk to one another so that the relevance and importance of their contributions could be understood. However, the lengthy time it takes to build collaborative relationships among different disciplinary scholars, the effort to create a common language and the cost to invest in it often result in less research funding ( Lindell et al, 2016).

## Driving forces across the Strait

In Hong Kong, Taiwan and China, various forces mobilised the initiatives of medical humanities. In Taiwan, medical humanities emerged in the 1990s along several trajectories, including the general education reform, physicians’ social involvement after the lifting of Martial Law and the medical education reform called for by returning physicians from the USA who formed the Joint Commission of Taiwan in 1999 for the improvement of healthcare quality by administering hospital accreditation ( Liu et al, 2004). In Hong Kong, medical humanities found its way to enter medical education through the undergraduate medical programme (MBBS) restructuring as part of the Hong Kong-wide reform of secondary and tertiary educational structures. The strength of the curriculum formation lay in its creation as a niche-based project after the three-year preparatory work that addressed the issue of the ‘neglected curriculum’ untaught in medical schools by asking questions, such as the following: ‘What makes us human?’ ‘What gives our lives meaning?’ ‘What is the nature of suffering?’ The work included site visits to institutions with successful medical humanities programmes, a needs survey among faculty staff and students, as well as hosting a conference to brainstorm on ideas and to draw on the experiences of overseas and local experts ( Chen et al, 2017). In China, medical humanities was first used as a discourse to guide health policies and good clinical practice. Around the year 2000, the merger of medical colleges and general research universities provided opportunities to develop medical humanities programmes through academic exchanges between the medical and the humanities faculties of the universities. The achievement could be exemplified by the establishment of the Institute of Medical Humanities at Peking University in 2008 and the virtual course in medical humanities created by Fudan University in 2015 ( Yun, 2017).

With the realisation of interdisciplinary teamwork surfacing in these three Chinese-speaking societies, reflections and challenges have begun to surface over the past decade. In Taiwan, for example, scholars report that ten years after the curricula were incorporated into official medical education, consensus on the real meaning of medical humanities has not yet been reached. The absence of a definition consensus has led to cognitive disparity among the faculty members, resulting in various expectations about curriculum outcomes and barriers to curriculum planning ( Wu et al, 2008). There have been insufficient communication platforms between clinical and humanities teachers. Additionally, medical humanities has suffered from exclusion from scholars who have placed more emphasis on clinical research ( Tsui et al, 2008). In China, scholars point out that the development of medical humanities has lagged behind the rapid growth of medical sciences. A major disadvantage to medical humanities is reflected on the disproportionate funding support granted by The National Science Foundation of China or the National Natural Science Fund of China ( Yun et al, 2017). In Hong Kong, medical humanities has been included in the Medical Council’s official document, Hong Kong Doctors, as one of the required core competencies for medical students to acquire, and the programme at The University of Hong Kong has thus continued to receive the medical faculty’s support ( The Medical Council of Hong Kong, 2017, p. 3-4).

## Hong Kong’s experience

In Hong Kong, as a curriculum initiative, the medical humanities curriculum in the MBBS programme at The University of Hong Kong (HKU) was envisioned to be an interdisciplinary endeavour and to have its development and delivery reflected in the multi-disciplinary composition and diverse expertise of the curriculum planning group and the teachers. The leaders of the MH initiative made a deliberate effort to include and recruit interested colleagues from all specialities and departments in the Faculty who were engaged in teaching medical students. Through a curriculum retreat and questionnaire survey, colleagues were identified and nominated by heads of department to be part of the inaugural medical humanities planning group (MHPG) convened in 2013. There were representatives from most teaching departments/units in the medical faculty from both basic science and clinical disciplines. Colleagues from the university-at-large in the fields of social sciences, fine arts, education and history, who were already known to members, were also invited resulting in a membership of 37. All of the MHPG members had research and teaching expertise in their own fields but none had prior experience teaching or working in medical humanities in an undergraduate medical education context. This enabled the group to develop fresh ideas from the ground up, bringing diverse perspectives to enrich the syllabus and the classroom learning.

Many of the learning activities in the MH curriculum are led concurrently by teachers from both the arts and from medicine which gives the visual impression of the interdisciplinary collaboration that underscores the interdisciplinary exploration of a particular topic. Interdisciplinary perspective emerging from interdisciplinary collaborative teaching remains a key ideal of the MH curriculum. For example, one of the threads in the MH curriculum examines the history of medicine in Hong Kong as it relates to why we practice medicine the way we do. ‘Narrative Reflections on Crisis’ was a three part exploration of this topic from three seemingly divergent yet intersecting perspectives. The historian introduced the concept of crisis, using plague (a defining event in the history of medicine in Hong Kong) as an example to focus on the various repercussions on doctors and society. The public health researcher extended the discussion beyond plague to look at other infectious disease crises to explore patterns in history, how history informs the present and future, and how health care progress also brings forth social and ethical issues. The primary care doctor looked at front line human emotion in times of crisis (individual crises as well as global crises) and how we experience it, are affected by it and respond to it. Together, the teachers co-created a cohesive and authentic interdisciplinary learning experience for students.

Another thread in the MH curriculum is culture and healing which examines the roles of culture and spirituality in healing. One of the learning activities is a workshop that introduces mindful practice as a means to be fully present and aware of self and surroundings with implications for patient care, as well as a skill to build personal resilience. This is led by a social scientist and a doctor, both of whom are active mindfulness practitioners who are able to bring in the scientific basis of mindful practice, the practical aspects and the relevance to medicine ( Wong et al, 2016). Another is a drama workshop led by a performance arts educator and a neurosurgeon, that forces students out of their comfort zone by having them dramatise a scene from a well-known play to examine doctors’ motivations and relationships with patients. The active engagement of both teachers reinforces the value of what may be perceived to be an irrelevant paedgogical approach (performance arts) by medical students and makes explicit its relevant to medical practice through a debriefing discussion. If such a session was led by a teacher from only one or the other discipline, the learning would not be nearly as compelling ( Chen et al, 2016).

In 2015, the hiring of a historian to join the core team under the medical humanities programme at The University of Hong Kong became the first cross-disciplinary academic appointment in a medical faculty in Hong Kong. In 2018, the first cohort of students under the compulsory and longitudinal medical humanities curriculum recently graduated from the six-year programme. The formation of an interdisciplinary group and subgroups for curriculum planning and teaching enabled powerful learning experiences for both teachers and students. However, the tendency was for these interactions to be rather superficial as most of the teachers are engaged in one-off teaching sessions. All the members of the MHPG are committed and enthusiastic volunteers in the MH program but are compelled by external academic pressures to limit the extent and depth of their involvement. The potential for true interdisciplinarity in the HKU context is truly promising but as yet unfulfilled.

Moreover, the challenges of medical humanities education are observed at another three levels. First, regarding the content, scholars have argued about the problems that might occur in implementing the Western style of medical education, including medical humanities, as a neo-imperialist product in the global context ( Bleakley 2015; Hooker et al, 2011). Whilst it might be easier to implement this style of teaching and learning, such as problem-based learning, it takes more effort to contextualise the content of medical humanities in response to local needs. Owing to the undergraduate degree structure, students generally do not recognise the significance of medical humanities until they enter their senior year. However, when they finally become aware of the curriculum relevance, they have no more time left for recollection or reflection. Second, concerning the maintenance of interdisciplinary teamwork, the number of participants from other faculties in the Medical Humanities Planning Group have varied over the years. Therefore, this situation requires efforts to maintain the connection between the medical humanities programme and scholars from other faculties. Few of them deliver lectures or conduct workshops on a regular basis. Third, as for faculty development, the teaching team has to rely on community partners and volunteer clinicians to undertake teaching and learning activities for more than 1,000 students. An effective faculty development plan is lacking, not only because of the absence of awareness, but also due to the little time available to squeeze the programme into the teachers’ busy schedules under the work conditions prevailing in Hong Kong.

## Humanities and social sciences scholars’ new identity

In April 2018, at the Annual Science, Technology and Society Conference in Tainan, Taiwan, humanities and social science scholars from various medical schools in Taiwan reflected on the challenges in designing and offering courses related to medical humanities. In the specially curated session, they brainstormed on their work from three aspects: the perpetually vague definition of medical humanities, the confused identities of non-scientific scholars working for medical schools and the difficulties in cross-disciplinary collaboration. The participants first pondered on their shifted identity from pure academics to active communicators across disciplines, attempting to reach out to clinical teachers and create dialogues to offer different perspectives on analysing health, whereas clinicians’ views were mostly limited to their own rationales. Whilst communicating with clinical teachers, they found that special skills are needed, particularly the ability to connect teams, to work across boundaries and to work in the environment they are not familiar with. Sadly, it was observed that few clinicians become the same communicators. They found clinicians too used to reducing critical thinking to measurable skillsets. However, with their new identity, the participants also perceived these crises as avenues to exercise their agency to inspire the design and the delivery of medical education, as well as take further steps to influence policy making.

Nevertheless, a larger problem lies in how higher education is imagined, operated and manipulated in the institutions where the scholars work. In the same session, they reflected on the tensions regarding the impartiality of performance reviews or criteria for promotion on the tenure tracks of humanities and social science scholars working in a biomedical-oriented environment. In Asian universities, journal metrics have created new imaginaries for ‘top universities’ and reshaped institutional behaviours ( Mok, 2007; Chou et al, 2013). Such patterns have resulted in the enlarged size of the science faculties, gradually edging out the space for humanities and social sciences. For example, teaching releases and sabbatical leaves are less likely to occur in medical schools, but these breaks provide the important research support required for time-consuming article writing. Without such support, it is difficult to conduct in-depth and contextualised research and transform the results into teachable content. In terms of research, humanities scholars and social scientists still have to focus on their original research output since it is less likely to publish interdisciplinary works. Regarding teaching, relying on non-contextualised materials is therefore inevitable. Teachers can only reduce lengthy debates on medical history and anthropology to the most basic concepts, without being able to upscale their teaching from the level of reflection to critical thinking ( Wu, 2017). Likewise, the pursuit of increasingly stringent metric-based performances in different university departments creates barriers to academic exchange among scholars who are not yet tenured. Additionally, with the often unquantifiable outcome of medical humanities education, the metric approach usually does not reflect the curricula’s achievements ( Gillis 2007). The resources are thus limited in supporting immeasurable programmes.

## Conclusion

In 1977, philosopher Ivan Illich (2011) commented on the restricted function of professions in society. In an interview, Illich remarked on the embodied practice of a physician, ‘They brought the patient to the hospital and, with their newly discovered diagnostic methods, they established a chart. They then treated the chart, they changed its parameters. When the chart was healthy, frequently without looking at the guy - I’m caricaturing, of course - they told him to put on his shoes and go home..’ ( Cayley, 2007, p. 141). This is the image of a physician whom medical humanities has been attempting to critique. In other words, this is the product that medical humanities education has been avoiding to create. However, according to Illich, constraints in present-day higher education reflect the reluctance to change the position taken by medical graduates as they start their careers. They are then expected to support the status quo, still portraying the role of professionals as agents of the elite class. It has been argued that the field of medical humanities has so far failed to challenge accepted and comfortable medical norms and assumptions, making little progress beyond tinkering around the edges of medical education ( Macnaughton, 2011).

In in present-day higher education, internationalisation and interdisciplinarity has become a common pursuit and has been written in their strategic planning objectives. Such phenomenon is more obvious in research-focus universities in Asia, where researchers find themselves less and less comfortable to live, talk and learn with people from different intellectual, disciplinary and methodological backgrounds. In the universities of Taiwan and Hong Kong and some of China’s leading universities, the development of medical humanities epitomises the conflicts between the two pursued values. For the past decade, scholars have called for ‘global medical humanities’ in response to the initiatives such as democratising medicine that incorporate global values and logics ( Bleakley, 2015). Identical appraisal of Asian curricula regarding their positions in the world, nonetheless, have not yet been critically conducted. It is time for developers of medical humanities, and more importantly, medical education at a higher level, to rethink and strategise the way that the teaching of medical humanities is positioned and delivered. Otherwise, the vision of medical humanities remains a castle in the air.

## Take Home Messages

• Medical humanities is by nature interdisciplinary despite the fact that levels and forms of interdisciplinarity might differ.
• Medical humanities programmes in Hong Kong, Taiwan and China were enabled by various social forces and the higher education reform around the millennium.
• Challenges of medical humanities programmes identified by humanities and social scientists scholars include: teachers’ altering identity, problem of content contextualisation, lack of communication among faculties and absence of faculty development.
• Asian universities’ practice of internationalisation could lead to changes in interdisciplinary workforce that obstructs the design and the delivery of medical humanities curricula.

## Notes On Contributors

Harry Yi-Jui Wu is Assistant Professor and Deputy-Director at Medical Ethics and Humanities Unit, Li Ka Shing Faculty of Medicine, The University of Hong Kong. He is also trained as a historian of medicine.

Julie Y. Chen is Associate Professor (Teaching) at Department of Family Medicine and Primary Care and Bao Institute of Medical and Health Sciences Education, Li Ka Shing Faculty of Medicine, The University of Hong Kong.

## References

[ref1] Albert M. Paradis E. and Kuper A. (2015) Interdisciplinary promises versus practices in medicine: The decoupled experiences of social sciences and humanities scholars. Social Science & Medicine. 126, pp.17–25. 10.1016/j.socscimed.2014.12.004 25500163

[ref2] Bleakley A. (2015) Medical Humanities & Medical Education: How the Medical Humanities can Shape Better Doctors. London and New York: Routledge.

[ref3] Cayley D. (2007) Ivan Illich in conversation. Toronto: House of Anansi Press.

[ref4] Chen J.Y. Yau L Loo N Wong A and Chan L.C. (2016), Fostering reflection-in-action through a performance arts workshop.in Peterkin A and Brett-MacLean P (eds.) Keeping Reflection Fresh: A Practical Guide for Clinical Educators. Kent: Kent University Press.

[ref5] Chen J.Y. Wu H. Y.-J. (2017) Medical Humanities.in Dent J. (eds.) Practical Guide for Medical Teachers. 5th Edition. ( Springer). pp.222–229.

[ref6] Chou C.P. Lin H.F and Chiu Y. (2013) The impact of SSCI and SCI on Taiwan’s academy: an outcry for fair play. Asia Pacific Education Review. 14:23–31. 10.1007/s12564-013-9245-1

[ref7] Fox D. (1985) Who we are: the political origins of the medical humanities. Theoretical Medicine and Bioethics. 6(3), pp.327–342. 10.1007/BF00489733 3904071

[ref8] Gillis C. M. (2007) Medicine and Humanities: Voicing Connections. Journal of Medical Humanities. 29(1), pp.5–14. 10.1007/s10912-007-9045-x 18064545

[ref9] Hooker C. and Noonan E. (2011) Medical humanities as expressive of Western culture. Medical Humanities. 37(2), pp.79–84. 10.1136/medhum-2011-010120 22114347

[ref10] Illich I. (2011) Disabling professions. New York: M. Boyars.

[ref11] Jones T. Blackie M. Garden R. and Wear D. (2017) The Almost Right Word. Academic Medicine. 92(7), pp.932–935. 10.1097/ACM.0000000000001518 28657553

[ref12] Lindell B. Dinnage R and Hua X. (2016) Interdisciplinary research has consistently lower funding success. Nature. 534, pp.684–687. 10.1038/nature18315 27357795

[ref13] Liu J.S. and Liu K.M. (2004) A preliminary study of discourses and practice on “medical humanities” in Taiwan’s medical education reform. Journal of Medical Education. 8(4), pp.371–384. (In Chinese).

[ref14] Macnaughton J. (2011) Medical humanities challenge to medicine. Journal of Evaluation in Clinical Practice. 17(5), pp.927–932. 10.1111/j.1365-2753.2011.01728.x 21851510 PMC4439737

[ref15] Mok KH (2007) Questing for internationalisation of universities in Asia: Critical reflections. Journal of Studies in International Education. 11(3–4), pp.433–454. 10.1177/1028315306291945

[ref16] The Literature, Arts and Medicine Database (LitMed) of NYU (1993) Humanities, Social Sciences & The Arts in Relation to Medicine & Medical Training. Available at http://medhum.med.nyu.edu/about( Accessed 28 of August, 2018).

[ref17] The Medical Council of Hong Kong (2017) Hong Kong Doctors. Available at: https://www.mchk.org.hk/english/publications/files/HKDoctors.pdf( accessed 28 August 2018).

[ref18] Tsui H.C. and Ho M.J. (2008) The Evolution of Medical Humanities Education in Taiwan: Qualitative Analysis by In-depth Interviews. Journal of Medical Education. 12(3), pp.133–141.

[ref19] Wear D. (2009) “The Medical Humanities: oward a Renewed Praxis. Journal of Medical Humanities. 30(4), pp.209–220. 10.1007/s10912-009-9091-7 19763797

[ref20] Wong R Wong V Chen J.Y. and Chan L.C. (2016) Reflective space: the medical library as a mindfulness sanctuary.in Peterkin A and Brett-MacLean P (eds.) Keeping Reflection Fresh: A Practical Guide for Clinical Educators. Kent: Kent University Press

[ref21] Wu C.H. Hsu H.H. & Ho M.J. (2008) The Medical Humanities Curriculum in Taiwan: Curriculum Survey and Student Interviews. Journal of Medical Education. 12(2), pp.107–17.

[ref22] Wu H. Y.-J. (2017) Six domains to develop critical medical humanities. The Clinical Teacher. 15(2), pp.93–97. 10.1111/tct.12713 28994514

[ref23] Yun X Guo J and Qian H. (2017) Preliminary thoughts on research in medical humanities. BioScience Trends. 11(2), pp.148–151. 10.5582/bst.2017.01085 28458331

