# Supplementary material for: Conundrum between internationalisation and interdisciplinarity: reflection on the development of medical humanities in Hong Kong, Taiwan and China
Source: MedEdPublish (2016). 2018 Sep 3;7:184. [Version 1] doi: 10.15694/mep.2018.0000184.1 (PMC10701838; doi:10.15694/mep.2018.0000184.1)
Supplement: Peer review history

# Reviewer report: Reviewer response for version 1

**DOI:** 10.21956/mep.19662.r29350

**Published:** 2018-09-07

**Authors:** Jonathan McFarland

**Affiliations:** 1 Sechenov University, Moscow

This review has been migrated. The reviewer awarded 5 stars out of 5 I wish to thank the authors for submitting this very important and relevant article which is an in-depth refection on the development of the medical humanities in Hong Kong, Taiwan and China. It is a timely contribution, as now is the moment to join forces and build ties on an international scale. I thoroughly enjoyed and learnt from the whole article but would like to highlight certain points. While discussing the Hong Kong experience the authors mention how teachers from the Arts are working hand in hand with those from medicine; this interdisciplinarity is critical, and mirrors what I have always believed to be the way forward. Along these lines I found some of the particular projects that had been put into practice fascinating, such as: using the history of medicine in Hong Kong to explain the specificities of the medicine practised there, the use of mindfulness and a drama workshop led by an arts educator and a neurosurgeon. I must say that I would be very interested to learn more about these particular projects as I am sure that they can be used in different cultural contexts around the world.What the article shows clearly is what progress has been made but also how far there is still to go, and we do need to work together to address these problems that are stopping the medical humanities make the REAL difference. And some of the problems are – the lack of an agreed definition of MH, the difficulties of cross-disciplinary collaboration, and the difficulties in measuring the effects of MH. These difficulties are there to be overcome and will be when we start to take note of and learn from experiences like the ones very well described in this article. For this reason alone, I would unhesitatingly recommend this article to all those interested in the field of the humanities in medical education. Thank you.

Reviewer Expertise:

NA

No decision status is available

# Reviewer report: Reviewer response for version 1

**DOI:** 10.21956/mep.19662.r29349

**Published:** 2018-09-04

**Authors:** Richard Hays

**Affiliations:** 1 James Cook University

This review has been migrated. The reviewer awarded 4 stars out of 5 Thank you for the invitation to review this paper. While it is rather heavy reading, as it takes a historical approach to the development of medical humanities in 'three Chinas', rather than describe the richness of the offerings. I know most about what happens in Hong Kong, where the Government mandates a component of language, history and literature in all university courses, and both medical schools add interesting content related to ethics, philosophy and the visual arts, although in different ways. The program at HKU is among the best I have seen, and the students appear to appreciate the broader base of the medical course. I would have awarded the extra star if the authors had included examples, or at least links to examples, of the material covered by medical students. Never-the-less, the paper is worth reading by those engaged in curriculum reform, because it shows how the leadership can be engaged in something a little broader than pure scientific knowledge and clinical skills. The paper may encourage development elsewhere of broader, multi-disciplinary approaches to providing medical students with some 'glue' to strengthen the necessary professional approach to applying modern science to health care problems encountered. After all, these problems present in people, who are more than a bunch of molecules, and medical humanities assist practitioners to understand both themselves and the patients they serve.

Reviewer Expertise:

NA

No decision status is available

# Reviewer report: Reviewer response for version 1

**DOI:** 10.21956/mep.19662.r29348

**Published:** 2018-09-03

**Authors:** Trevor Gibbs

**Affiliations:** 1 AMEE

This review has been migrated. The reviewer awarded 5 stars out of 5 As someone who is both interested in the Humanities and has also been heavily involved in all forms of teaching in mainland China and Hong Kong, I was naturally drawn to this paper; I was not disappointed as I read it. It is well written, although at times I felt a little difficult to understand and took a while to grasp the authors' meaning, well structured and well referenced. As with some other papers in this theme, I felt it took a slightly different look at the teaching of the Humanities. It brought to light the difficulties we now experience with defining the Humanities, how it can be content and context specific and the difficulty experienced in its interdisciplinary nature ( I would prefer to use the word interprofessional). For many schools globally the humanities are restricted to the teaching of the history of the country, its social development and sometimes the background to its religious culture without any relationship to health and disease. To others it is clearly the relationship between the arts and health and how the arts can engender a different approach to caring. As we battle with whether the Humanities should or should not be part of our standard curricula, papers such as these are intrinsic to our learning, and would suggest that this paper is read by all involved in curriculum development.I look forward to reading other papers from these authors and their educational unit, especially their intentions on how to take forward their Humanities teaching and learning.

Reviewer Expertise:

NA

No decision status is available
